# Sintilimab Plus Bevacizumab Biosimilar Versus Sorafenib as First-Line Treatment for Unresectable Hepatocellular Carcinoma: A Cost-Effectiveness Analysis

**DOI:** 10.3389/fphar.2022.778505

**Published:** 2022-02-09

**Authors:** Ye Peng, Xiaohui Zeng, Liubao Peng, Qiao Liu, Lidan Yi, Xia Luo, Sini Li, Liting Wang, Shuxia Qin, Xiaomin Wan, Chongqing Tan

**Affiliations:** ^1^ Department of Pharmacy, The Second Xiangya Hospital of Central South University, Changsha, China; ^2^ PET-CT Center, The Second Xiangya Hospital of Central South University, Changsha, China; ^3^ Xiangya School of Nursing, The Second Xiangya Hospital of Central South University, Changsha, China

**Keywords:** sintilimab, bevacizumab biosimilar, cost-effectiveness, hepatocellular carcinoma, markov model

## Abstract

**Objective**: The ORIENT-32 clinical trial revealed that sintilimab plus bevacizumab biosimilar significantly improved the median progression-free survival and median overall survival (OS) compared with sorafenib. This analysis evaluated the cost-effectiveness of sintilimab plus bevacizumab biosimilar as a first-line treatment for unresectable hepatocellular carcinoma from the Chinese perspective of healthcare system.

**Materials and methods**: A Markov model with three mutual health states was constructed to evaluate the economic outcome of sintilimab plus bevacizumab biosimilar. The model cycle was 21 days, and the simulation time horizon was a lifetime. The output parameters of the model were the total cost, life-year (LY), quality-adjusted LY (QALY), and incremental cost-effectiveness ratio (ICER). Sensitivity analyses were conducted to assess the robustness of the results.

**Results**: The base-case results found that sintilimab plus bevacizumab biosimilar provided an improvement of 1.27 QALYs and 1.84 LYs compared with sorafenib, and the ICER was $23,352/QALY. The hazard ratio for OS had the greatest influence on the ICER. The probability of sintilimab plus bevacizumab biosimilar was 85% at willingness-to-pay thresholds of $30,552/QALY.

**Conclusion**: The findings of this analysis suggested that sintilimab plus bevacizumab biosimilar was a cost-effective first-line therapy for patients with unresectable hepatocellular carcinoma.

## 1 Introduction

Primary liver cancer is the third leading cause of cancer-related deaths in the world, with an estimated 830,000 deaths in 2020 (Sung et al., 2021). Its main type is hepatocellular carcinoma (HCC), which accounts for 75–85% of all cases (Sung et al., 2021). The predominant causative factor of HCC is hepatitis B virus (HBV). The new cases of HBV infection in China account for about half of the global cases (Chen et al., 2016). The burden of HCC in China is high, and the prognosis of patients with HCC is poor (Ren et al., 2021).

Sorafenib, as the first-line standard care for advanced HCC, has moderate efficacy and limited survival benefits (Llovet et al., 2008; Cheng et al., 2009; Kudo et al., 2018). However, recent research progress has shown that the combination of anti–programmed cell death-1 (PD-1) antibodies and anti-angiogenic drugs may become a potential first-line therapy for HCC (Ren et al., 2021). The ORIENT-32 clinical trial evaluated the efficacy and safety of sintilimab, a selective anti-PD-1 antibody (Gao et al., 2020), in combination with bevacizumab biosimilar in the treatment of unresectable HCC (Ren et al., 2021). The results revealed that sintilimab plus bevacizumab biosimilar significantly improved the median progression-free survival (PFS) and median overall survival (OS) when compared with sorafenib. Sintilimab plus bevacizumab biosimilar also had a lower incidence of grade 3–5 adverse events (AEs) than sorafenib (29 vs. 47%). Therefore, the sintilimab plus bevacizumab biosimilar therapy seems to be an attractive option as a first-line treatment for unresectable HCC. The present analysis evaluated the cost-effectiveness of sintilimab plus bevacizumab biosimilar as a first-line treatment for unresectable HCC from the perspective of the Chinese healthcare system.

## 2 Materials and Methods

### 2.1 Analytical Overview

**FIGURE 1:**
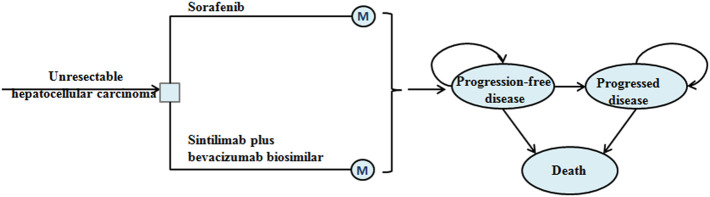
Model structure of a Markov model combining the decision tree. M, Markov.

This analysis assumed that the target population was patients with unresectable HCC who had not previously received systemic therapy, which is consistent with the ORIENT-32 trial (Ren et al., 2021). A Markov model with three mutual health states was constructed to evaluate the economic outcome of sintilimab plus bevacizumab biosimilar or with sorafenib in unresectable HCC (Figure 1). The model cycle was 21 days, and the simulation time horizon was a lifetime. The output parameters of the model were total cost, life-year (LY), quality-adjusted LY (QALY), and incremental cost-effectiveness ratio (ICER). All costs were adjusted to be expressed in US dollars, and the exchange rate was 1 US dollar = 7.07 Chinese yuan (June 2020). The costs and outcomes were adjusted using an annual discount rate of 3% (Sanders et al., 2016). The willingness-to-pay (WTP) threshold was proposed to be three times that of the per-capita gross domestic product of China in 2020 (US $30,552) (Murray et al., 2000). Statistical analysis was performed using TreeAge Pro (TreeAge Software, Williamstown, MA) and R.

### 2.2 Clinical Data Inputs

**FIGURE 2:**
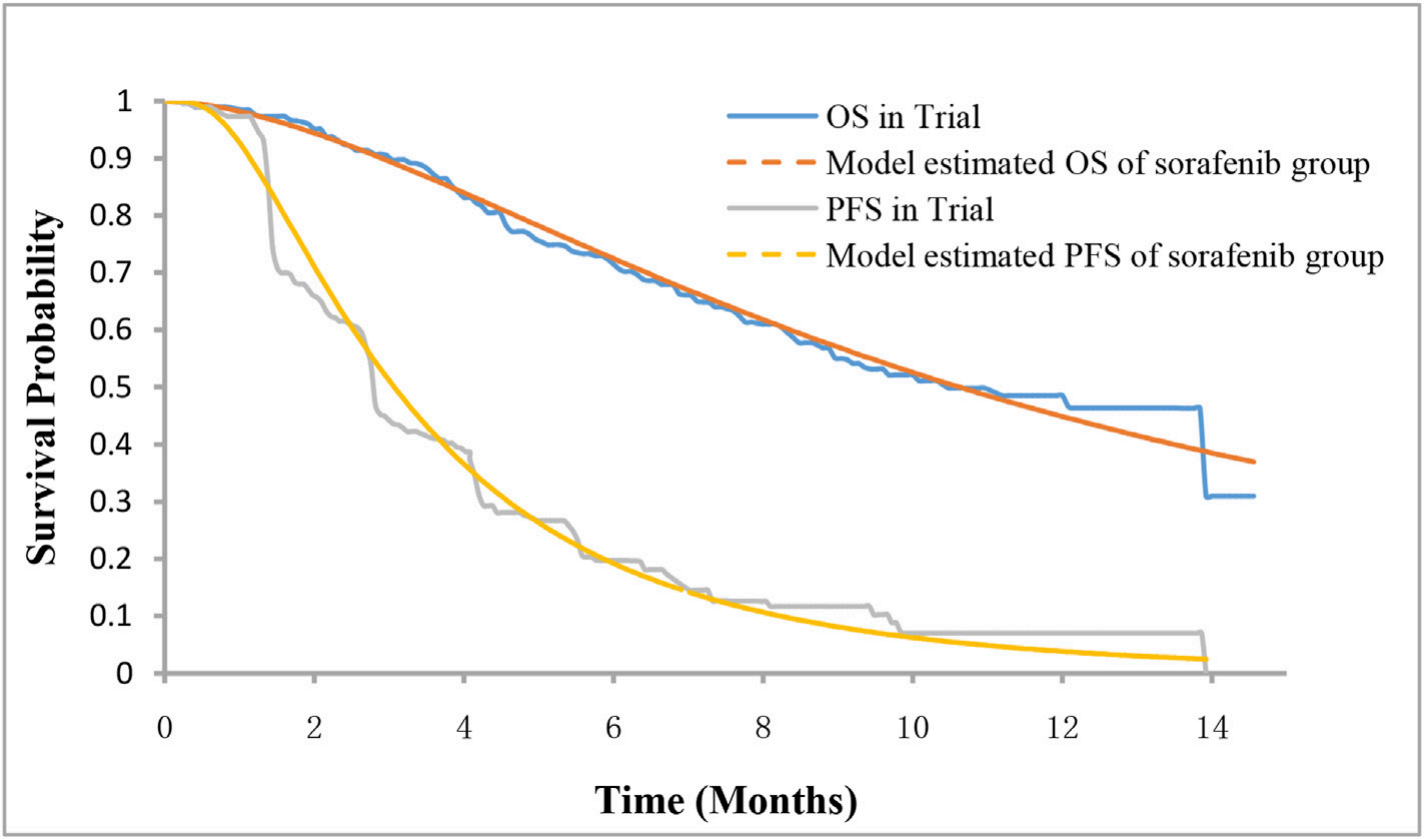
Survival curves for model and the phase III trial. PFS, progression-free survival; OS, overall survival.

Among the three health states, the probability of staying in the progression-free disease (PFD) state was based on the PFS curve. The transition probability from PFD state to progressed disease (PD) state was estimated by the difference between OS and PFS curves. The probability of death was obtained by subtracting the area under the OS curve from 1. The PFS and OS data of patients in the sorafenib arm were derived from the results of the ORIENT-32 trial (Ren et al., 2021), and the data beyond the clinical trial time horizon were extrapolated using the method described by Guyot et al. (2012). First, the data points in the survival curves were gathered using GetData Graph Digitizer, and then the parametric survival functions of Weibull, log-logistic, log-normal, exponential, Gompertz, and generalized gamma were fitted. The final survival functions were selected for the sorafenib arm based on the Akaike information criterion. The transition parameters and proportions are shown in Table 1 and Figure 2. The PFS and OS data for the sintilimab plus bevacizumab biosimilar arm were calculated based on the hazard ratios (HRs) of sintilimab plus bevacizumab biosimilar versus sorafenib reported in the ORIENT-32 trial. After the disease progressed, the data on patients receiving subsequent treatment were derived from the ORIENT-32 trial (Ren et al., 2021). The key clinical inputs are demonstrated in Table 1.

**TABLE 1:** Key Model Inputs.

Parameter	Expected value (range)	Distribution	References
Clinical input			
Survival model for sorafenib			
Log-normal model for PFS	mu = 1.121355, sigma = 0.7702185		Ren et al. (2021)
Log-logistic model for OS	lambda = 0.01822555, gamma = 1.694566		Ren et al. (2021)
HR for PFS associated with sintilimab–bevacizumab biosimilar vs. sorafenib	0.560 (0.460–0.700)	Log-normal	Ren et al. (2021)
HR for OS associated with sintilimab–bevacizumab biosimilar vs. sorafenib	0.570 (0.430–0.750)	Log-normal	Ren et al. (2021)
Proportion receiving subsequent treatment			
Sorafenib	0.470 (0.376–0.564)	Beta	Ren et al. (2021)
Sintilimab–bevacizumab biosimilar	0.290 (0.232–0.348)	Beta	Ren et al. (2021)
Sorafenib arm: incidence of Grade ⩾3 AEs			
Increased aspartate aminotransferase	0.05 (0.040–0.060)	Beta	Ren et al. (2021)
Decreased platelet count	0.03 (0.024–0.036)	Beta	Ren et al. (2021)
Increased blood bilirubin	0.03 (0.024–0.036)	Beta	Ren et al. (2021)
Hypertension	0.06 (0.048–0.072)	Beta	Ren et al. (2021)
Palmar–plantar erythrodysesthesia syndrome	0.12 (0.096–0.144)	Beta	Ren et al. (2021)
Sintilimab–-bevacizumab biosimilar arm: incidence of Grade ⩾3 AEs			
Increased aspartate aminotransferase	0.020 (0.016–0.024)	Beta	Ren et al. (2021)
Decreased platelet count	0.08 (0.064–0.096)	Beta	Ren et al. (2021)
Increased blood bilirubin	0.05 (0.040–0.060)	Beta	Ren et al. (2021)
Hypertension	0.14 (0.112–0.168)	Beta	Ren et al. (2021)
Palmar–plantar erythrodysesthesia syndrome	0 (0–0)	Beta	Ren et al. (2021)
Utility input			
Utility of PFD	0.760 (0.610–0.910)	Beta	Su et al. (2021)
Utility of PD	0.680 (0.540–0.820)	Beta	Su et al. (2021)
Disutility due to AEs			
Grade 1 and 2	0.010 (0.008–0.020)	Beta	Su et al. (2021)
Grade 3 and higher	0.160 (0.110–0.204)	Beta	Su et al. (2021)
AEs cost, $/event			
Increased aspartate aminotransferase	87 (70–105)	Gamma	Wen et al. (2021)
Decreased platelet count	1,054 (843–1,265)	Gamma	Wen et al. (2021)
Increased blood bilirubin	114 (91–136)	Gamma	Wen et al. (2021)
Hypertension	1.35 (1.08–1.62)	Gamma	Wen et al. (2021)
Palmar–plantar erythrodysesthesia syndrome	34 (27–40)	Gamma	Wen et al. (2021)
Drug cost, $/per cycle			
Sintilimab	804 (643–965)	Gamma	Local charge
Bevacizumab biosimilar	1,465 (1,172–1758)	Gamma	Local charge
Sorafenib	790 (632–948)	Gamma	Local charge
Test	352 (282–423)	Gamma	Li et al. (2021)
Pembrolizumab	5,069 (4,055–6,082)	Gamma	Local charge
Regorafenib	1,747 (1,397–2,096)	Gamma	Local charge
BSC	357 (286–428)	Gamma	Li et al. (2021)

OS, overall survival; PFS, progression-free survival; HR, hazard ratio; AEs, adverse events; PFD, progression-free disease; PD, progressed disease; BSC, best supportive care.

### 2.3 Cost and Utility Inputs

Only direct medical costs such as drugs cost, testing cost, AEs management cost, and best supportive treatment were considered (Table 1). The costs were reported in 2020 US dollars and adjusted to 2020 value using the consumer price index (National Bureau Of Statistics Of China, 2021).

Based on the ORIENT-32 trial (Ren et al., 2021), patients in the sintilimab plus bevacizumab biosimilar arm received intravenous injections of 200 mg sintilimab and 15 mg/kg bevacizumab biosimilar every 3 weeks. Patients in the sorafenib arm received 400 mg of sorafenib orally twice daily. To calculate the dose of bevacizumab biosimilar, a typical Chinese patient weighing 60 kg was assumed (Wen et al., 2021). Treatment in both arms continued until disease progression or unacceptable toxicity, or the sintilimab plus bevacizumab biosimilar arm was treated for up to 24 months. After disease progression, subsequent therapy was received in 29% of patients in the sintilimab plus bevacizumab biosimilar arm and 47.0% of patients in the sorafenib arm (Ren et al., 2021). Tyrosine kinase inhibitors (regorafenib) and immune agents (pembrolizumab) were hypothesized to be the second-line options of the two arms based on the ORIENT-32 trial (Ren et al., 2021). All cost information are shown in Table 1.

The utility values of PFD and PD states related to HCC were assumed to be 0.76 and 0.68 (Su et al., 2021), respectively, based on the published cost-effectiveness analysis of patients with HCC. The disutility values related to AEs and the costs related to managing grade ≥3 AEs were also included in this analysis (Table 1) (Su et al., 2021; Wen et al., 2021).

### 2.4 Base-Case Analysis

The ICER was the incremental cost for each additional QALY between the sintilimab plus bevacizumab biosimilar arm and the sorafenib arm. It was assumed to be cost-effective when the ICER was below the prespecified WTP threshold ($30,552/QALY) (Neumann et al., 2014). Sensitivity analyses were conducted to assess the robustness of the results. One-way sensitivity analysis was performed on all key parameters. The range of each parameter was set based on the published literature or assumed to be a 20% change in the base-case value (Table 1). Monte Carlo simulations with 10,000 iterations were performed in the probabilistic sensitivity analysis. Pre-specified distributions for all key parameters are shown in Table 1. The cost-effectiveness acceptability curve was used to indicate the possibility of being considered cost-effective at different WTP levels.

## 3 Results

### 3.1 Base-Case Analysis

The base-case results of the cost-effectiveness analysis are shown in Table 2. The sintilimab plus bevacizumab biosimilar treatment provided 2.30 QALYs and 3.30 overall LYs, with an accompanying cost of $59,018. The sorafenib treatment provided 1.03 QALYs and 1.47 LYs at a cost of $29,351. The ICER for sintilimab plus bevacizumab biosimilar vs sorafenib was estimated to be $23,352/QALY.

**TABLE 2:** Base case results.

Results	Sorafenib	Sintilimab plus bevacizumab biosimilar	Incremental
LYs	1.47	3.30	1.84
QALYs	1.03	2.30	1.27
Total cost, $	29,351	59,018	29,668
ICER, $			
Per LY			16,149
Per QALY			23,352

LYs: life-years; QALYs: quality-adjusted LYs; ICER: incremental cost-effectiveness ratio.

### 3.2 Sensitivity Analysis

**FIGURE 3:**
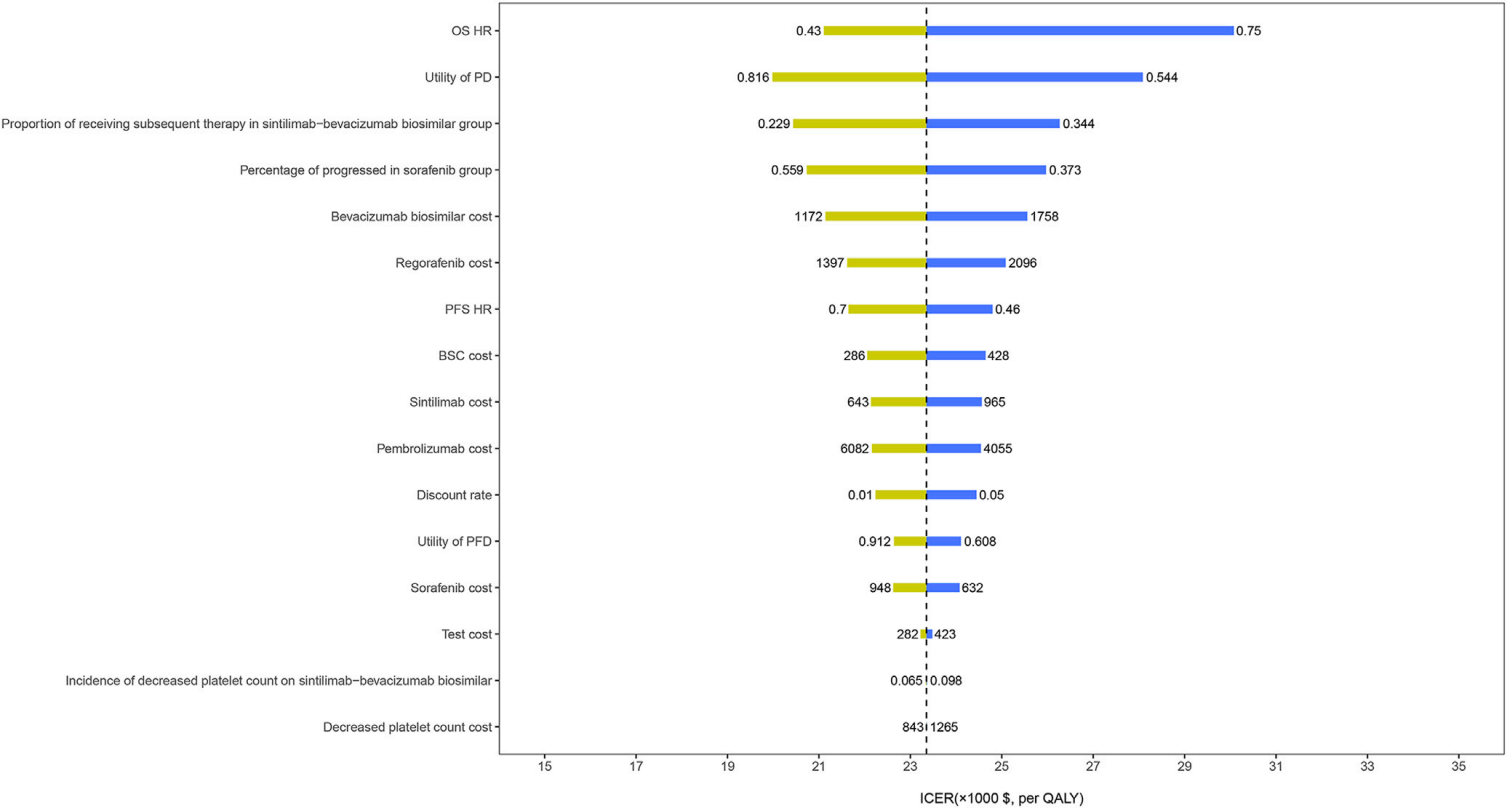
One-way sensitivity analyses. OS, overall survival; HR, hazard ratio; PD, progressed disease; PFS, progression-free survival; BSC, best supportive care; PFD, progression-free disease; QALYs, quality-adjusted life-years; ICER, incremental cost-effectiveness ratio.

**FIGURE 4:**
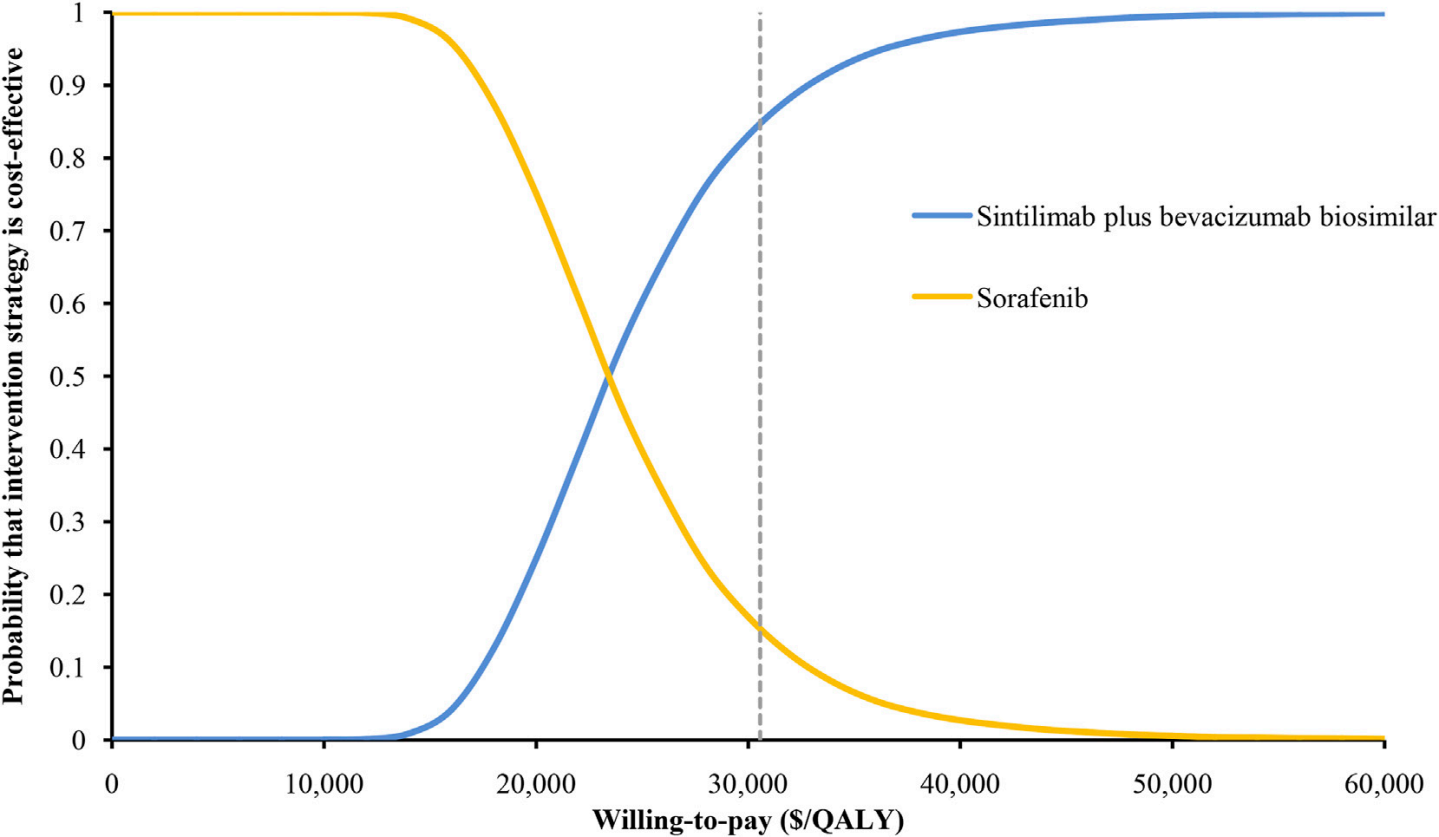
Cost-effectiveness acceptability curves. QALYs, quality-adjusted life-years.

The results of the one-way sensitivity analysis showed that the HR for OS had the greatest influence on the ICER, followed by the utility of PD, the proportion receiving subsequent treatment, and the cost of bevacizumab biosimilar (Figure 3). However, these parameters varied within a range, and the ICER has always remained below the threshold of $30,552/QALY. The results of the probabilistic sensitivity analysis were displayed by the cost-effectiveness acceptability curve. The probability of sintilimab plus bevacizumab biosimilar being cost-effective increased as the WTP thresholds increased (Figure 4). Sintilimab plus bevacizumab biosimilar has an 85% probability of being cost-effective compared with sorafenib at the WTP thresholds of $30,552/QALY.

## 4 Discussion

We conducted a cost-effectiveness analysis of sintilimab plus bevacizumab biosimilar versus sorafenib in the first-line treatment of unresectable HCC. The ICER for sintilimab plus bevacizumab biosimilar was estimated as $23,352/QALY versus sorafenib. The probabilistic sensitivity analysis indicated that the probability of sintilimab plus bevacizumab biosimilar was 85% at the WTP threshold of $30,552/QALY.

Recently, the immune checkpoint inhibitor (ICI) combination with PD-1 antibody and cytotoxic T-lymphocyte–associated protein-4 (CTLA-4) antibody has been proved to be effective for advanced HCC. The Phase 1/2 clinical trial (CheckMate 040) from 31 centers in 10 countries showed that nivolumab plus ipilimumab had manageable safety, promising objective response rate, and durable responses for patients with advanced HCC previously treated with sorafenib (Yau et al., 2020). In 2020, the U.S. Food and Drug Administration approved nivolumab in combination with ipilimumab for the treatment of patients with HCC previously treated with sorafenib (Saung et al., 2021). However, there is currently no relevant and sufficient large-scale phase III clinical trial data. Once the data is available, we will conduct a pharmacoeconomic evaluation on it.

The choice of the comparator in the model is an important issue in cost-effectiveness analysis. According to the treatment guidelines for HCC, there are several first-line therapy options for unresectable HCC (Bureau of Medical Administration National Harmon Health Commission of the People’s Republic of China, 2020). This makes the choice of comparator a challenge. Lenvatinib, another first-line targeted therapy approved for unresectable HCC has not been included in this study. But we speculate that the ICER of this group might be lower than the estimate in our study due to similar clinical efficacy and higher cost when compared with sorafenib (Kudo et al., 2018). Due to the lack of head-to-head data, other immune checkpoint inhibitors have not been included in the evaluation, such as atezolizumab plus bevacizumab, which also have favorable survival benefits as a first-line treatment therapy for unresectable HCC (Finn et al., 2020). Two economic evaluations in China comparing sorafenib with atezolizumab plus bevacizumab have found sorafenib to be cost-effective (Wen et al., 2021; Hou and Wu 2020).

In our study, sintilimab plus bevacizumab biosimilar was superior to sorafenib, which is different from a previous study on atezolizumab and bevacizumab. The present study suggests that the cost-effectiveness of the ICI combination therapy might not be due to the therapeutic effect but to biosimilar itself. Bevacizumab biosimilars are similar to the reference bevacizumab in terms of efficacy and safety (Yang et al., 2019; Zhang et al., 2019), but the price of bevacizumab biosimilars is much lower than the price of the original drug. Therefore, this will increase the probability that sintilimab combined with bevacizumab is cost-effective. In recent years, value-based cancer treatment has become a hot topic in oncology. The development of biosimilar products provides patients with affordable alternative therapy.

Some studies have evaluated the cost-effectiveness of immunotherapy in HCC. Almost all studies have shown that immunotherapy is unlikely to be a cost-effective option compared to sorafenib, although the ICERs reported by these studies vary (Hou and Wu 2020; Chiang et al., 2021a; Su et al., 2021; Wen et al., 2021; Zhang et al., 2021). Four of these studies that used clinical data from the IMbrave150 trial evaluated the economics of atezolizumab plus bevacizumab in the first-line treatment of HCC from the perspective of the United States (Chiang et al., 2021a; Su et al., 2021; Wen et al., 2021; Zhang et al., 2021). The study by Wen et al. (2021) also evaluated the economics of atezolizumab plus bevacizumab from the perspective of China, with an ICER of $145,546.21/QALY (Wen et al., 2021). A China-based study showed that atezolizumab plus bevacizumab was unlikely to be cost-effective compared with sorafenib as a frontline treatment for patients with unresectable HCC, with an ICER was $64,613/QALY (Hou and Wu 2020). Based on the clinical data of the KEYNOTE-240 trial, the study by Chiang et al. (2021b) conducted a cost-effectiveness analysis of pembrolizumab as a second-line treatment for HCC in the United States. The results indicated that pembrolizumab was unlikely to be cost-effective. To our knowledge, our study was the first to analyze the cost-effectiveness of sintilimab plus bevacizumab biosimilar in the first-line treatment of HCC. There is some strength to this analysis that is worth highlighting. First, this analysis evaluated the economic outcomes of sintilimab plus bevacizumab biosimilar in the treatment of unresectable HCC through economic modeling methods and the synthesis of the latest evidence. Monotherapy blockade of PD-1 alone or combined with other regimens is becoming an attractive option for the treatment of unresectable HCC (Mahipal et al., 2019). Furthermore, this study addressed the unmet needs of the economic evaluation related to advanced HCC.

This analysis also has limitations. First, the efficacy and safety parameters of the model are essentially based on the results of the ORIENT-32 trial. Any bias in this trial may inevitably affect the cost and effectiveness. Second, based on the Kaplan–Meier PFS and OS data reported in the ORIENT-32 trial, the long-term survival data beyond the observation time was extrapolated by the fitting of the parametric distribution. This may lead to uncertainty in the output of the model, although the model and observation data have been validated. Third, data availability and assumptions have also led to the limitation of our analysis. The use of ±20% variation in range in the sensitivity analysis to explain the uncertainty may not reflect the true situation. However, it had also been used as an acceptable boundary in similar studies (Kohn et al., 2017). Fourth, the management cost of grade 1–2 AEs has not been considered in this analysis. However, the sorafenib arm had a higher incidence of grade 1–2 AEs than the sintilimab plus bevacizumab biosimilar arm, which may cause ICER to be lower than the estimated value of our study. Moreover, the results of the one-way sensitivity analysis showed that the costs associated with AEs were minor. This study showed that sintilimab plus bevacizumab biosimilar was a cost-effective first-line therapy for patients with unresectable HCC. These findings may help clinicians make optimal decisions for the treatment of HCC.

## Data Availability

The original contributions presented in the study are included in the article/Supplementary Material, further inquiries can be directed to the corresponding authors.
